# Development and validation of measures to evaluate adolescents' knowledge about human papillomavirus (HPV), involvement in HPV vaccine decision-making, self-efficacy to receive the vaccine and fear and anxiety

**DOI:** 10.1016/j.puhe.2017.02.006

**Published:** 2017-06

**Authors:** A.S. Forster, K.A. McBride, C. Davies, T. Stoney, H. Marshall, K. McGeechan, S.C. Cooper, S.R. Skinner

**Affiliations:** a Department of Behavioural Science and Health, UCL, London, WC1E 6BT, UK; b Centre for Health Research, Western Sydney University, NSW, Australia; c Discipline of Child and Adolescent Health, Children's Hospital Westmead, University of Sydney, NSW, Australia; d Telethon Kids Institute, University of Western Australia, Perth, Australia; e Vaccinology and Immunology Research Trials Unit, Women's and Children's Health Network and Robinson Research Institute and School of Medicine, University of Adelaide, Adelaide, Australia; f School of Public Health, University of Sydney, Sydney, Australia; g Department of Community Health and Social Sciences, CUNY Graduate School of Public Health and Policy, New York, USA

**Keywords:** Papillomavirus vaccines, Health knowledge, Attitudes, Practice, Surveys and questionnaires, Reproducibility of results, Adolescent, Decision-making

## Abstract

**Objectives:**

We describe the development and validation of measures of human papillomavirus (HPV)/HPV vaccination knowledge, fear/anxiety about vaccination, involvement in HPV vaccine decision-making, and self-efficacy with regard to getting the vaccine, designed to evaluate the efficacy of an intervention to affect these domains (collectively termed the HAVIQ: HPV Adolescent Vaccine Intervention Questionnaire).

**Study design:**

Literature search, cognitive interviews and cross-sectional survey.

**Methods:**

A literature search identified existing items that were modified for the present measures. Experts reviewed draft measures for face and content validity. Cognitive interviews with adolescents were also used to assess content validity. Adolescents completed the measures and an internal reliability analysis of each measure was performed.

**Results:**

The four experts concurred that the measures had face validity. Cognitive interviews identified items requiring refinement. Content validity was examined with ten experts and was deemed acceptable. There were 1800 adolescents who completed the measures; Cronbach's alpha was >0.6 for three of the four measures. The four final measures are brief, comprising 25 items in total.

**Conclusions:**

The measures are robustly developed and validity-tested. The HAVIQ may be used in research settings to evaluate adolescents' knowledge and experiences of the process of HPV vaccination in a school-based vaccination programme.

## Highlights

• We validated measures of HPV knowledge, fear, involvement in decision-making and self-efficacy.
• Experts confirmed the face and content validity of the measures.
• Adolescents helped refine the measures to assure content validity.
• Three of the four measures had good internal reliability.

## Introduction

A national school-based programme in Australia offers the human papillomavirus (HPV) vaccine to adolescent males and females in the first year of secondary school (year seven or eight, depending on jurisdiction, age 11–14 years), free-at-the-point-of-receipt.^1^, ^2^ A quadrivalent HPV vaccine is used in the Australian programme (given in three doses at zero, one and six months). This vaccine is designed to prevent infection and disease caused by HPV types 6 and 11, which are responsible for >90% of anogenital warts, and HPV genotypes 16 and 18, which worldwide are responsible for approximately 50% of precancerous lesions and 70% of cancerous lesions of the cervix.^3^, ^4^, ^5^ HPV infections are also responsible for an increasing subset of head and neck cancers.^6^ Three-dose vaccination coverage for girls in Australia is relatively high, at 73% for most recent estimates, with corresponding coverage for males just over ten percentage points lower.^7^ These rates of vaccine coverage have resulted in impressive reductions in HPV 16 and 18 prevalence^8^ and in high-grade cytological lesions.^9^

In the Australian programme, the jurisdictional public health authority responsible for implementation of the programme provides a hard copy of a parent information brochure and consent form (usually via the adolescent who is given it to take home from school). In general, active parental consent is required for vaccination. Previously no information was provided to the adolescents themselves, with perhaps an assumption that parents would convey appropriate information to their adolescent. However, our previous research has identified that parents do not often speak to their adolescent about the HPV vaccine, for reasons of lack of confidence in their own knowledge and feelings of embarrassment where discussion might involve the sexually transmitted nature of the virus.^10^, ^11^, ^12^ Our data have identified a lack of basic knowledge and understanding among both adolescent girls and their parents regarding HPV, the vaccine and cervical cancer.^12^ In 2014, as part of a Federal government health department initiative, information resources for schools have been made available via a website (www.hpv.gov.au). It is unclear how well these resources have been taken up by schools, as yet, or their impact on students. Indeed, our research has shown that there have been challenges with effective inter-sectorial collaboration between health and education government sectors in the delivery of vaccination around issues pertaining to the student experience in particular.^13^

Fear and anxiety regarding the vaccine has also been reported, which may in part be attributed to this lack of knowledge and the presence of myths and rumours.^14^, ^15^ This fear and anxiety has been found to not only affect uptake^16^, ^17^ but also completion of the vaccine series.^18^ It also has a detrimental effect on the vaccination process in schools, with individual vaccinations taking longer while adolescents are calmed.^14^

There is some evidence that involving adolescents in the decision to get the vaccine can affect uptake.^19^ Informed decision-making can be empowered by education, which may facilitate adolescents' having a voice in whether they get the vaccine or not.^20^ We found that while some adolescents were involved in this decision, a significant proportion were not.^21^ Shared decision-making was particularly hindered by parent/adolescent discomfort with talking about sex together. Australian girls have said that they would be more involved in the decision to be vaccinated if they were equipped with knowledge about the vaccine.^12^

Theoretical models of health behaviour suggest a role for self-efficacy (one's belief in one's ability to perform a certain behaviour, akin to perceived behavioural control) in explaining why individuals do or do not engage in health behaviours and these models have been applied in the context of HPV vaccination.^22^ We hypothesised that self-efficacy to be involved in the decision-making process would be predictive of actual involvement in the decision and expressed anxiety and fear. Therefore we also considered self-efficacy to be an important concept to consider.

On the strength of these data and reasoning, we developed an educational intervention for adolescents aged 11–14 years, due to be offered HPV vaccination (as part of a larger intervention also targeting parents and vaccination programme organisational issues),^20^, ^23^ that aimed to effect change in four domains hypothesised to be related to student experience of vaccination and vaccination uptake: (1) knowledge about HPV and the HPV vaccine; (2) adolescents' involvement in the decision-making process; (3) fear and anxiety associated with the vaccine; and (4) self-efficacy in receiving the vaccine.^20^ It is necessary to have a robust measure of these domains to evaluate the efficacy of interventions, such as our own, designed to change these domains. A literature review identified that past research had used items similar to those required for use. However, no scale fully met our needs: a brief measure, validated for use with young adolescents (aged 11–14 years) that could be practically administered within the context of a mass school vaccination programme before and after the educational intervention and acceptable to all types of school. In this paper we describe the development and validation of four short measures designed to detect change in these domains, known collectively as the HPV Adolescents Vaccination Intervention Questionnaire (HAVIQ).

## Methods

The development and validation of the measures occurred in a number of stages: item identification, measure development and assessment of face validity, content validity and internal reliability. Item identification and scale development are described in full in this methods section, with the results of the validation and reliability analyses reported in the Results section.

### Item identification

We identified potential items and existing measures of each domain in the context of HPV vaccination, as well as other preventive health behaviours, from a detailed desk-based rapid review of the literature. Existing items were modified or items created for the present measures.

#### Knowledge

Very many studies have measured knowledge or awareness of HPV/HPV vaccination (e.g.^24^, ^25^, ^26^, ^27^). There were appropriate questions in all of these studies that could have been used in the knowledge measure, but the question content was most comprehensively covered in a well-developed scale for adults that was being validated at the time of this research.^28^ We therefore based the majority of the items on that scale.

#### Self-efficacy

Two studies were identified that measured self-efficacy in the context of getting the HPV vaccine^25^, ^26^ and two items from these studies were adapted for our self-efficacy measure. A number of other studies addressed self-efficacy in relation to other preventive health behaviours. One study in particular examining the psychometric properties of the Self-Efficacy Questionnaire for Children included items that were relevant for adolescents^29^ and two of these were rephrased so that they were appropriate for our self-efficacy measure. We also rephrased five items that were psychometrically tested in phobic situations.^30^ One final item was rephrased from the Measurement of Self-Efficacy and Externality scale so that it was relevant to HPV vaccination.^31^

#### Fear and anxiety

Two studies were found that sought to assess fear and/or anxiety in relation to HPV vaccination. One study used questions to evaluate general feelings towards needles^26^ and two items from this study were adapted for our fear and anxiety measure. Another study sought to assess girls' psychological response to a leaflet containing information about HPV infection and vaccination.^24^ These authors used a short six-point form of the Spielberger State Trait Anxiety Inventory (STAI) which has been previously assessed and found to generate results that were comparable to using the full form of the STAI.^32^ The short version of the STAI was used as a basis for formulating HPV vaccine-specific items.

#### Involvement in decision-making

We were aware of a study, at the time unpublished^33^ that measured HPV vaccination decision-making in university students. We rephrased several items from this study so that they were suitable for adolescents in a school-based setting. Other items were generated by the research team based on the findings of two studies that had considered factors that were deemed relevant to involvement in decision-making.^26^, ^34^

### Measure development

Using the items identified in the literature review, we collectively drafted a preliminary version of the measures. Existing items sourced from the literature were reworded where necessary and, where gaps in the domains to be measured were identified, new items were developed by the research team to ensure that all facets of the intervention were covered. There were 37 questions in the initial item pool, 15 of which were developed by the research team. We ensured that the measures were of an appropriate reading level for our intended participants (11–14 year old adolescents) by conducting a readability assessment with Flesch's statistic in Microsoft Word. All items were checked by the research team for ambiguity, use of jargon and length and double barrelled questions avoided as they can be difficult to answer with a single response.^35^ Items were worded in either direction of the domain being measured to preclude generalised responses to the items, as this can limit validity.^36^, ^37^

Likert-type scale response categories were used for the involvement in decision-making and fear/anxiety measures as dichotomous ‘yes’/‘no’ responses can result in a loss of information^35^ and can restrict respondents' answers.^38^ Five to nine response categories are usually recommended for Likert scales,^35^ so a five-point scale was used with the options ‘strongly disagree’, ‘disagree’, ‘neither agree nor disagree’, ‘agree’ and ‘strongly agree’. For the knowledge measure, we opted for a five-point scale.

It is recommended that self-efficacy is measured with a 100-point scale, with participants choosing a number to indicate how confident they are (one being ‘not confident at all’ and 100 being ‘completely confident’).^39^ However, we also considered that a shorter (five-point) Likert-type scale could be simpler to complete as it would be consistent with the other measures. We opted to test both response scales for ease of use.

### Assessment of face validity

The draft HAVIQ was reviewed by four international experts in the field of school-based HPV vaccine delivery and psychosocial aspects of uptake to check for possible omissions. Recommendations for improvement were also sought.

### Assessment of content validity

#### Cognitive interviews with adolescents

We conducted cognitive interviews with adolescent females, aged 12 and 13 years old to ascertain whether there were problems with any items, to understand their thoughts on the measures in general and whether they had any suggestions for improvements. These interviews were conducted prior to HPV vaccination being offered to boys in Australia. A further function was to test the appropriateness of the response options. Cognitive interviews were conducted in two waves, with second wave participants commenting on items that had been revised as a result of the first wave of cognitive interviews. Girls were recruited through informal networks, with cognitive interviews conducted at the girls' homes and with both parents and adolescents providing informed consent. Approval for these interviews was obtained from the Children's Hospital at Westmead Research Ethics Committee.

#### Expert review

Following the cognitive interviews, we conducted another expert review as an additional measure of content validity. The aim of the expert review was to assess if the items in each measure were relevant and whether the four measures were adequately represented by the selected items.^40^, ^41^ Ten national/international experts with expertise in HPV vaccination (including paediatricians, psychologists, epidemiologists, a nurse and a general practitioner) were asked to rate each item as being ‘essential’, ‘useful but not essential’ or ‘not necessary’ as well as provide any comments on each measure. Four of these experts had previously commented on the face validity of the measures.

Content validity was calculated using Lawshe's formula.^42^ Lawshe proposes that each expert's ‘*essential*’ rating be used to calculate the level of agreement for each item's inclusion resulting in a content validity ratio (CVR) for each measure. The formula for this is: *CVR* = (*n*_*e*_ − *N*/*2*)/(*N*/*2*) with *n*_*e*_ being the number of ‘*essential*’ ratings per item and *N* being the number of expert panellists. CVR greater than 0.62 for ten experts is considered acceptable.

### Assessment of internal reliability

Internal reliability was assessed by asking adolescents to complete the HAVIQ. Adolescents were recruited as part of a large randomised controlled trial. Recruitment methods have been described fully elsewhere.^23^ In brief, participants were male and female adolescents in their first or second year of high school (year 8 or 9) who were due to be offered the HPV vaccine as part of the school-based programme. The study obtained full ethical approval from Western Australia's Department of Health Human Research Ethics Committee and South Australia's Women's and Children's Hospital Human Research Ethics Committee. Participants were adolescents recruited from the 19 schools in the control arm of the trial; a stratified random sample of 40 schools across two Australian States.^23^ Schools were diverse in type (co-educational/single sex and independent, government and catholic schools). Students completed the HAVIQ in school prior to being offered HPV vaccination. Cronbach's alphas were calculated for each measure using SPSS, along with an item-correlation matrix and Cronbach's alphas if items were to be deleted from each measure.

## Results

We began development of the HAVIQ in 2010 and completed face and content validity assessments in 2011. Reliability was completed in early 2016, with data collected in 2013 and 2014. The preliminary version of the HAVIQ had a Flesch's reading score of 66.1 and a Flesch-Kincaid grade level of 6.2, indicating that it was suitable for adolescents of our targeted age group.

### Face validity

All four experts concurred that the measures should effectively capture any changes in knowledge, fear/anxiety and self-efficacy and would also quantify adolescents' involvement in the decision-making process.

### Content validity—cognitive interviews

Several suggestions made by adolescents during cognitive interviews (*n* = 5, all female) included simplifying items by removing words and rewording questions. One example of this was to change the item ‘HPV can be caught by engaging in sexual activity’ to ‘HPV can be caught through sexual activity’.

Sometimes adolescents answered items by considering what their peers might think, rather than answering specifically about their beliefs. We adjusted items to clarify this (i.e. ‘having to get a needle can be upsetting’ was extended to ‘having a needle can be upsetting to me’).

Four out of the five adolescents preferred the 100-point self-efficacy response option because it allowed them to be more accurate, said that it ‘made more sense and was easier to understand’ and was ‘more like a percentage’ (which is used by their teachers when marking exams).

The measure underwent further cognitive interviewing with two adolescents to check the modifications made following the first round of cognitive interviews. Three items were removed as a result of the second round of interviews as their revised wording meant that the items had lost their meaning or were ambiguous.

### Content validity—expert review

Initial calculations from our expert panel (*n* = 10) revealed that the content validity index (CVI) was low for each measure (Table 1), none of which met the minimum CVI of 0.62 that is required for the ratings of 10 experts.

**Table 1:** Content validity index: progressive analyses.

Analysis stage	Knowledge	Self-efficacy	Fear and anxiety	Decision-making
First	0.40	0.04	0.42	0.38
Final	0.87	0.72	0.67	0.63

We therefore examined questions with a particularly low number of *essential* ratings and thus a low CVR, to establish whether they could be discarded to improve the overall CVI of each measure. The CVIs for each measure were then recalculated in a second analysis after dropping several items, including five items from the decision-making measure, three from the knowledge measure, five from the self-efficacy measure and four from the fear/anxiety measure. This improved the measures of CVIs so that all were greater than the minimum requirements for content validity (Table 1).

Qualitative comments from the experts were also concurrently reviewed. On the whole, feedback was positive, with few suggestions for improvements or rewording to the items. Unsurprisingly, the majority of comments related to items that were removed during the CVI analysis stage.

### Internal reliability

The HAVIQ was completed by 1800 male (*n* = 982) and female (*n* = 817) adolescents (1 = gender unknown). For the fear/anxiety and involvement in decision-making measures, ‘strongly disagree’ responses were scored 1, through to 5 for ‘strongly agree’. With hindsight the study team decided that knowledge should have been measured using the response options ‘true’, ‘false’ or ‘don't know’. For this reason, we recoded the knowledge measure response options as ‘strongly disagree/disagree’, ‘neither agree nor disagree’ and ‘strongly agree/agree’, as proxy measures of ‘true’, ‘false’ and ‘don't know’. For example, for the question ‘HPV is very rare’, the responses ‘strongly disagree’ and ‘disagree’ were akin to ‘true’. Each self-efficacy item was scored out of 100. For each measure, item totals were summed to give a measure total.

The Cronbach's alphas for the knowledge measure (alpha = 0.60), fear/anxiety measure (alpha = 0.79) and self-efficacy measure (alpha = 0.79) were acceptable. Item total statistics suggested that the deletion of items from these three measures would not remarkably alter the Cronbach's alpha; item correlation was adequate and not indicative of multicollinearity. The alpha for the involvement in decision-making measure was poor (alpha = 0.35), however item correlation was good with no evidence of multicollinearity and removing items would not improve the Cronbach's alpha.

### The final measure

The final HAVIQ, comprises 25-items assessing adolescents' knowledge about HPV/HPV vaccination, their involvement in the vaccine decision-making process and their fear/anxiety and self-efficacy with regard to the vaccine with four separate measures (see Supplementary Material for the measure).

## Discussion

This measure of adolescents' knowledge about HPV/HPV vaccination, their involvement in the vaccine decision-making process and their fear/anxiety and self-efficacy with regard to the vaccine was deemed to have face and content validity, and three of the four measures had acceptable internal reliability. The HAVIQ has been used to assess the efficacy of a school-based intervention aiming to maximise uptake of HPV vaccination,^20^, ^23^ with the separate measures having specific research applications in the evaluation of adolescents' knowledge and experiences of the process of HPV vaccination in a school-based vaccination programme.

An understanding about the transmission of HPV as well as the development of anogenital warts, and HPV-related cancers is particularly pertinent to adolescents, given that they are vulnerable to HPV infection and subsequently at risk for cervical, anal, penile, vulvar, vaginal, oropharyngeal cancers and anogenital warts.^43^, ^44^, ^45^ Knowledge about the HPV vaccine, HPV virus and its relationship with these cancers and anogenital warts is considered fundamental in ensuring informed vaccination decision-making.^46^, ^47^, ^48^ Evidence suggests that improved knowledge may facilitate increased adolescent involvement in immunisation decisions, reduce fear about getting vaccines, increase the likelihood that vaccination decisions are informed and their attitudes to vaccination are favourable.^12^, ^14^, ^15^, ^20^ It is important to be able to measure these domains in adolescents so that interventions to improve such domains can be evaluated. The four separate measures developed in this paper have research applications in the evaluation of adolescents' knowledge and experiences of the process of HPV vaccination in a school-based vaccination programme.

The study is not without limitations. The cognitive interviews were conducted with a limited number of interested adolescents, so their perceptions of the measure items may not reflect those of a more general population of adolescents. The decision-making measure did not have an acceptable level of internal reliability suggesting this measure could benefit from further modification or the individual items considered separately. However, the international experts concurred that the HAVIQ should assess what it was aiming to. With hindsight we would also recommend rewording the knowledge measure response options to ‘true’, ‘false’ and ‘don't know’. Since the development of this measure, an alternative scale measuring HPV knowledge in adults has been published, so other approaches to assessing knowledge may be considered.^28^ It would be useful to conduct a test-retest reliability of the measures. Although participants who completed the HAVIQ for the internal reliability analysis also completed the HAVIQ at additional time points, it was not possible to test responsiveness as participants would have been offered the HPV vaccine between data collection time points, so one might have expected their responses to change. However, to our knowledge, the HAVIQ is the only group of measures, designed specifically for young adolescents being offered HPV vaccination in a school programme and tested in this age group.

The HAVIQ is a robustly developed and psychometrically tested set of measures of adolescents' knowledge about HPV/HPV vaccination, involvement in vaccine decision-making and their fear/anxiety and self-efficacy with regard to the vaccine. The HAVIQ is designed to evaluate the efficacy of a HPV educational intervention designed to effect change in adolescents' knowledge and experiences of the process of HPV vaccination in a school-based vaccination programme, but could be used to evaluate interventions aimed at improving any of these domains among adolescents.

## Author statements

### Acknowledgements

The authors wish to thank Dr Mary McCaskill and the staff of the Emergency Department, The Children's Hospital at Westmead, Sydney, Australia, for assistance in recruitment of adolescents this project.

### Ethical approval

The internal validity study obtained ethical approval from Western Australia's Department of Health Human Research Ethics Committee and South Australia's Women's and Children's Hospital Human Research Ethics Committee. Approval for the content validity study was obtained from the Children's Hospital at Westmead Research Ethics Committee.

### Funding

This work was supported by the National Health and Medical Research Council Project Grant (NHMRC Project Grant 1026765). The work was also supported by GSK (who manufacture a HPV vaccine) and CSL (who distribute the HPV vaccine in Australia) through investigator led research grants (to SR Skinner and S Cooper), which provided funds to incentivise participation in the cognitive interviews, and to subsidise research assistant salaries. AS Forster is supported by a Cancer Research UK Research Travel Award [C49896/A21285] and a Cancer Research UK Cancer Prevention Fellowship [C49896/A17429]. H Marshall is supported by a NHMRC fellowship [APP1084951]. None of the funders or study sponsor had any role in the study design, collection, analysis and interpretation of data, in the writing of the report or the decision to submit the article for publication.

### Competing interests

TS was a principal investigator on GSK sponsored clinical trials for Cervarix, for which her institution received funding, a member of Asia Pacific Study Follow-up Committee for Studies Zoster (GSK), for which she has received honoraria and received travel support from bioCSL, Merck and GSK to attend and/or present at scientific meetings and conferences. HM is a member of vaccine advisory boards for Wyeth and GSK and her institution has received funding for investigator led research from Novartis, GSK and Sanofi Pasteur. She has received travel support from Pfizer, GSK and bioCSL to present scientific data at international meetings. SC and SRS received investigator driven research grant funding from GSK and bioCSL to develop and pilot educational materials used in this study. SRS was also a principal investigator on GSK sponsored clinical trials for Cervarix, for which her institution received funding. bioCSL, Merck and GSK are all HPV vaccine manufacturers. Gardasil (bioCSL/Merck) is currently funded for use in the Australian national HPV vaccination program.

### Contributions

KA Mahendran and SC Cooper developed the questionnaire and assessed the measure for content and face validity, with input from SR Skinner. AS Forster, SR Skinner and K McGeechan performed the internal reliability analysis. SR Skinner assisted in the validation study concept development and, with SC Cooper and KA Mahendran, developed the school-based education intervention being tested with HAVIQ. KA Mahendran and AS Forster wrote the manuscript. T Stoney and H Marshall were responsible for recruitment of adolescents to the HPV.edu study, whose data were used in the internal reliability analysis. SR Skinner, SC Cooper, K McGeechan and C Davies contributed to the manuscript development.
