# Supplementary material for: Development and validation of measures to evaluate adolescents' knowledge about human papillomavirus (HPV), involvement in HPV vaccine decision-making, self-efficacy to receive the vaccine and fear and anxiety
Source: Public Health. 2017 Jun;147:77–83. doi: 10.1016/j.puhe.2017.02.006 (PMC5476903; doi:10.1016/j.puhe.2017.02.006)
Supplement: Supplementary file 1 [file mmc1.doc]

**Decision-making**

Please indicate how much you agree with each statement.

|  | Strongly disagree | Disagree | Neither agree nor disagree | Agree | Strongly agree |
| --- | --- | --- | --- | --- | --- |
| My parents made the decision about having the HPV vaccine | □ | □ | □ | □ | □ |
| I did not really think much about the decision to have the HPV vaccine | □ | □ | □ | □ | □ |
| I feel that I was involved in deciding whether to have the HPV vaccine | □ | □ | □ | □ | □ |
| My friends’ decisions about the HPV vaccine influenced my decision | □ | □ | □ | □ | □ |
| I feel that I made the decision about whether to have the HPV vaccine myself | □ | □ | □ | □ | □ |
| My religious beliefs influenced my decision about the HPV vaccine | □ | □ | □ | □ | □ |
| I strongly value my health | □ | □ | □ | □ | □ |
| Prevention of diseases and infections is important to me | □ | □ | □ | □ | □ |

**Skills Inventory (Certainty)**

Rate your degree of confidence by recording a number from 0 to 100 using the scale given below:

| Cannot do at all | Moderately certain can do | Highly certain can do |
| --- | --- | --- |
| 0 10 20 30 40 50 60 70 80 90 100 | | |

| I am confident that I can receive all three doses of HPV vaccine | Confidence (0-100) : ______ |  |
| --- | --- | --- |
| I feel comfortable talking to my parents/caregivers about whether to have the HPV vaccine | Confidence (0-100) : ______ |  |
| I feel comfortable asking the nurses any questions I may have before receiving my HPV vaccination | Confidence (0-100) : ______ |  |
| I know what to expect when I have my HPV vaccination | Confidence (0-100) : ______ |  |
| I can cope with any pain I may experience when I have the vaccination | Confidence (0-100) : ______ |  |

**Feelings toward vaccination**

Please indicate how much you agree with each statement.

|  | Strongly disagree | Disagree | Neither agree nor disagree | Agree | Strongly agree |
| --- | --- | --- | --- | --- | --- |
| Having to get a needle can be upsetting to me | □ | □ | □ | □ | □ |
| I expect that the HPV vaccinations will be very painful | □ | □ | □ | □ | □ |
| I am not afraid of getting vaccinations | □ | □ | □ | □ | □ |
| I am worried about side effects of the HPV vaccine | □ | □ | □ | □ | □ |
| I feel tense when I hear other girls talking about the HPV vaccination | □ | □ | □ | □ | □ |
| I am concerned I may get cervical cancer in the future | □ | □ | □ | □ | □ |

**Knowledge**

Please indicate how much you agree with each statement.

|  | Strongly disagree | Disagree | Neither agree nor disagree | Agree | Strongly agree |
| --- | --- | --- | --- | --- | --- |
| Males cannot get HPV | □ | □ | □ | □ | □ |
| Women vaccinated against HPV need to get Pap smears (checks for cervical cancer) when they are older | □ | □ | □ | □ | □ |
| HPV can be caught through sexual activity | □ | □ | □ | □ | □ |
| HPV is very rare | □ | □ | □ | □ | □ |
| HPV can cause cervical cancer | □ | □ | □ | □ | □ |
| The HPV vaccine protects against all types of cervical cancer | □ | □ | □ | □ | □ |
